# A methyl esterase from *Bifidobacterium longum* subsp. *longum* reshapes the prebiotic properties of apple pectin by triggering differential modulatory capacity in faecal cultures

**DOI:** 10.1111/1751-7915.14443

**Published:** 2024-05-09

**Authors:** Inés Calvete‐Torre, Carlos Sabater, Nerea Muñoz‐Almagro, Ana Belén Campelo, F. Javier Moreno, Abelardo Margolles, Lorena Ruiz

**Affiliations:** ^1^ Group of Functionality and Ecology of Beneficial Microorganisms (MicroHealth) Dairy Research Institute of Asturias (IPLA‐CSIC) Villaviciosa Asturias Spain; ^2^ Health Research Institute of Asturias (ISPA) Oviedo Asturias Spain; ^3^ Group of Chemistry and Functionality of Carbohydrates and Derivatives Institute of Food Science Research, CIAL (CSIC‐UAM), Universidad Autónoma de Madrid Madrid Spain; ^4^ Dairy Research Institute of Asturias (IPLA‐CSIC) Villaviciosa Asturias Spain

## Abstract

Pectin structures have received increasing attention as emergent prebiotics due to their capacity to promote beneficial intestinal bacteria. Yet the collective activity of gut bacterial communities to cooperatively metabolize structural variants of this substrate remains largely unknown. Herein, the characterization of a pectin methylesterase, BpeM, from *Bifidobacterium longum* subsp. *longum*, is reported. The purified enzyme was able to remove methyl groups from highly methoxylated apple pectin, and the mathematical modelling of its activity enabled to tightly control the reaction conditions to achieve predefined final degrees of methyl‐esterification in the resultant pectin. Demethylated pectin, generated by BpeM, exhibited differential fermentation patterns by gut microbial communities in in vitro mixed faecal cultures, promoting a stronger increase of bacterial genera associated with beneficial effects including *Lactobacillus*, *Bifidobacterium* and *Collinsella*. Our findings demonstrate that controlled pectin demethylation by the action of a *B. longum* esterase selectively modifies its prebiotic fermentation pattern, producing substrates that promote targeted bacterial groups more efficiently. This opens new possibilities to exploit biotechnological applications of enzymes from gut commensals to programme prebiotic properties.

## Graphical abstract

The activity of a purified pectin methylesterase, BpeM, from *Bifidobacterium longum* subsp. *longum*, was experimentally validated through demetylation of highly methoyxylated apple pectin. The mathematical modelling of its activity enabled to tightly control the reaction conditions to achieve predefined final degrees of methyl‐esterification (DM) in the resultant pectin. Besides, demethylated pectin generated by BpeM, exhibited differential fermentation patterns by gut microbial communities in in vitro mixed fecal cultures promoting a stronger increase of bacterial genera associated with beneficial effects.

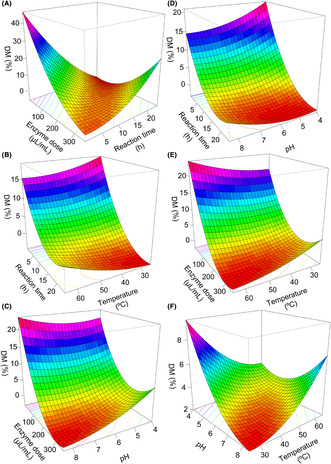

## INTRODUCTION

Prebiotics are substrates that are selectively utilized by host microorganisms conferring a health benefit (Gibson et al., 2017). Traditionally, prebiotics have been associated with the ability to promote the growth of members of the genus *Bifidobacterium*. Bifidobacteria encompasses several species frequently found in the human gastrointestinal tract (Alessandri et al., 2021), and includes a few species that have been granted the Qualified Presumption of Safety (QPS) status (EFSA, 2007). Among these, *Bifidobacterium longum* is one of the most abundant species in the human gut (Derrien et al., 2022; Milani et al., 2017) with a well‐documented scientific evidence on their activities (Hidalgo‐Cantabrana et al., 2017; Wong et al., 2020).

Classical prebiotics include a few well‐recognized categories of non‐digestible complex carbohydrates, such as human‐milk oligosaccharides (HMOs), galacto‐oligosaccharides (GOS), inulin and fructo‐oligosaccharides (FOS), as well as lactulose. However, in recent years, the concept of prebiotics has expanded to other types of poly‐ and oligosaccharides, mainly of plant origin, such as xylan, arabinoxylan and pectin (Calvete‐Torre et al., 2023; Sabater et al., 2021). Pectin is an appealing class of emergent prebiotic due to its capacity to promote beneficial microbial groups including lactobacilli, bifidobacteria and other groups of next‐generation probiotics such as *Faecalibacterium praustnizii*, *Eubacterium eligens* or certain *Bacteroides* species; and to trigger a number of health‐promoting effects (Bang et al., 2018; Beukema et al., 2020; Calvete‐Torre et al., 2022; Chung et al., 2017; Elshahed et al., 2021; Hussein, 2022; Lordan et al., 2020). Pectin is a natural macromolecular polysaccharide and one of the main constituents of plant primary cell walls. Chemically, pectin harbours a galacturonic acid backbone, which can alternate with linked‐rhamnose units bearing different side chains composed by various sugar moieties (Dranca et al., 2020). It can also include esterification with different moieties such as methyl or acetyl groups, resulting in a wide array of different pectin structures, that result in varied physical–chemical and biological properties (Beukema et al., 2020, 2021; Fan et al., 2020).

Pectin metabolism by intestinal bacteria has mainly been focused on the study of glycosidases (GHs) (El Kaoutari et al., 2013; Luis et al., 2018). Notably, products resulting from the action of GHs on plant polysaccharides can be further metabolized by accompanying members of the microbiota that take advantage foraging on the released oligosaccharides to complete the degradation of the substrates (Feng et al., 2018; Ndeh & Gilbert, 2018). Such cooperative networks are strongly dependent on the metabolic complementarity of coexisting microbiota members, and the specific polysaccharide structure (Payling et al., 2020). In addition to GHs, another group of Carbohydrate‐Activate Enzymes (CAZy), the carbohydrate esterases (CEs), contribute to pectin metabolization through catalysing the esterification or de‐esterification of substituted saccharides. CEs can act on a variety of residues decorating the sugar chains, such as fenolics, acetic and methyl esters. CEs are the first enzymes to act on complex polysaccharides, rendering glyosidic bonds accessible for further metabolization by GHs (Fries et al., 2007). Their encoding genes are generally located in gene clusters for polysaccharide metabolism, as it occurs in *Bacteroides*, a model gut bacterium with the ability to degrade a wide range of polysaccharide structures (PULs; Polysaccharide Utilization Locus) (Luis et al., 2018; Pereira et al., 2021; Robinson et al., 2017). However, in certain cases the genes required for complex polysaccharides metabolization may be scattered across a genome, rather than organized in PULs, as it occurs with the genes required for starch degradation in *Ruminococcus bromii* (Ze et al., 2015), or with those required for glycogen degradation in *Bifidobacterium* species (Esteban‐Torres et al., 2023). Among the enzymes required for polysaccharides utilization, pectin methylesterases from some intestinal microorganisms have been characterized (Duan et al., 2020; La Rosa et al., 2023; Ndeh et al., 2017). However, the role played by CEs in shaping the prebiotic properties of pectin and hence in affecting the metabolization patterns of the enzymatically modified substrates by gut microbial communities is unknown. In this work, we aim to contribute to unravel the role of a predicted pectin methylesterase from *B. longum* subsp. *longum*, a non‐pectinolytic gut commensal species, in shaping pectin fermentation pattern by mixed faecal cultures through the targeted modification of pectin.

## EXPERIMENTAL PROCEDURES

### Cloning of *bpeM* from *Bifidobacterium longum* in *Lactotococcus lactis* and purification of the enzyme

Total DNA was obtained from *Bifidobacterium longum* LMG13197 using the GenElute Bacterial Genomic kit (Sigma‐Aldrich, St. Louis), following the manufacturer's instructions. The structural gene of *bpeM* (Gene ID: 69577261, NCBI accession code: BLLJ_RS00045 pectinesterase family protein [*Bifidobacterium longum* subsp. *longum* JCM 1217], https://www.ncbi.nlm.nih.gov/gene/69577261/) was subsequently amplified by PCR enabling in‐frame cloning into the pNG8048E expression vector (Table S1) (Kloosterman et al., 2006). The PCR product was purified, *Bsp*HI and *Xba*I digested, and ligated with the *Nco*I/*Xba*I‐digested pNG8048E vector, resulting in pNBpeM. The plasmid was transformed into *L. lactis* NZ9000 electrocompetent cells by using the methods described previously (De Ruyter et al., 1996), and transformants were screened by restriction analysis of the plasmid. Correct cloning was verified by DNA sequencing.

For enzyme purification, *L. lactis* NZ9000 containing pNBpeM was grown in M17 (Difco) + 0.5% glucose (M17G) at 30°C. The O/N culture was inoculated (1% inoculum) in 1 litre of pre‐warmed to 30°C M17G, and cells were grown to an optical density at 600 nm of about 0.5. In order to trigger the transcription of *bpeM* from the *nisA* promoter, nisin (Sigma, St Louis, MO, USA) was added to the culture at a final concentration of 2 ng/mL and the cells were incubated for 90 min at 30°C, which resulted in an OD600 of about 1.2. Cells collection, lysis and protein purification were performed by using Ni‐NTA agarose (Qiagen) columns as previously described (Marcos‐Fernández et al., 2021). For Western blot analysis, SDS‐PAGE gels were transferred and immobilized on polyvinydilene fluoride (PVDF) membranes (GE Healthcare, Madrid, Spain) as previously described (Marcos‐Fernández et al., 2021). PVDF membranes were blocked and incubated following the manufacturer's instructions of the Penta·His HRP Conjugate Kit (Qiagen).

### Enzymatic activity modelling

To study pectin de‐esterification process using purified BpeM, 43 different test conditions were designed following a factorial experimental design with four independent variables (Table S2). The independent variables and ranges selected were: reaction time (0.23–24 h), pH (4.0–8.4), temperature (25–65°C) and enzyme dose (2.33–357.0 μL/mL pectin). All the reactions were conducted with an enzyme solution containing 352 μg/mL of the purified protein. The response variable studied was degree of methylesterification (DM) (%). Reactions at pH of 4.0 or 5.3 were done in 50 mM sodium acetate buffer; while those conducted at pH of 6.2; 7.1 and 8.4 were done in 50 mM sodium phosphate buffer.

De‐esterification reactions were conducted on pectin from apple pomace from Perico variety, obtained according to previously described procedures through ultrasound assisted extraction in a water‐batch during 30 min (Calvete‐Torre et al., 2021). Pectin employed presented a molecular weight of 732 kDa and a DM of 69% as previously reported (Calvete‐Torre et al., 2021). Reactions were set up at 20 mg/mL pectin in 1 mL tubes, incubated in Eppendorf ThermoMixer C, and reactions were stopped at 100°C during 5 min. Samples were lyophilized and stored at −80°C until their analyses. DM of the resultant pectin was determined through Fourier transform infrared spectroscopy (FTIR) analysis in the *Servicio Interdepartamental de Investigación* of Universidad Autónoma de Madrid (SIdI‐UAM), according to Calvete‐Torre et al. (2021) (Figure S1A–D). The experimental design and its analysis by response surface methodology (RSM) were computed on R v.4.2.2. using “DoE” and “rsm” R packages.

### Ethics statement and faecal donors

Ethics approval for this study was obtained from the Bioethics Committee of CSIC (Consejo Superior de Investigaciones Científicas) and from the Regional Ethics Committee for Clinical Research (Servicio de Salud del Principado de Asturias; no. 2020.278) in compliance with the Declaration of Helsinki. All donors provided written informed consent for their faecal matter to be used for the experiments, in fully compliance with the approved guidelines and regulations.

Faecal samples were provided by six healthy donors (aged between 20 and 50 years; 4 males and 2 females). They had not received any antibiotic treatment the 6 months prior to the sample collection, had no knowledge of having consuming pre‐ or probiotic supplements in the past 6 months, and were not suffering from any infectious diseases at the time of sample collection. Samples were collected and immediately transported to the laboratory where they were processed within 1 h from collection.

### Batch faecal fermentations

Native pectin obtained from apple pomace (DM 69%) and the same pectin de‐esterified through the action of the BpeM and presenting either 12%, 22%, or 42% of DM were subjected to faecal batch fermentation by using samples from healthy donors (*n* = 6). Since pectin is generally not very well fermented, and in order to increase our chances of incorporating pectin utilizing bacteria in the inoculums, independent duplicate faecal batch fermentations were inoculated with pooled faecal samples (two independent faecal pools combining three faecal samples each). Faecal pools for the inoculums were prepared weighing the same quantity of three freshly collected faecal samples and were homogenized together immediately prior to inoculation. The fermentations were performed in an anaerobic hood using the medium described in Sánchez‐Patán et al. (2015), containing as only carbon sources the substrates to be tested at a final concentration of 0.5% (w/v) as previously described (Calvete‐Torre et al., 2022). Fermentations were kept in the anaerobic chamber, with gentle stirring to prevent sediments precipitation, at 37°C. Samples were collected at times 0, 8 and 24 h, for DNA extraction and short‐chain fatty acids (SCFAs) analysis.

### DNA extraction, high‐throughput 16S rRNA sequencing and bioinformatics analysis of the faecal bacterial populations

DNA was extracted using the Power Soil ProKit (Qiagen) as described in Calvete‐Torre et al. (2022). Partial 16S rRNA sequencing was performed on 30 samples, corresponding to fermentations with five different conditions (pectin samples with four different DM values and a control fermentation without substrate addition), and two different faecal pool inocula, collected at three different time points along a 24 h fermentation period. The V3–V4 region was sequenced as previously described in the Sequencing Facilities of *Instituto de Parasitología y Biomedicina ‘López Neyra’* (Calvete‐Torre et al., 2022), by using the primers 16S ProV3V4 forward (CCTACGGGNBGCASCAG) and 16S ProV3V4 reverse (GACTACNVGGGTATCTAATCC). Sequence reads were quality filtered using a personalized script of QIIME2 v.2021.8 software and bioinformatic analysis of sequence data was performed on R v.4.2.2. following the computational pipeline of Calvete‐Torre et al. (2022). Briefly, 16S rRNA sequences were clustered in amplicon sequence variants (ASVs) and were classified to the lowest possible taxonomic rank using SILVA 138 (Callahan et al., 2016; Quast et al., 2013). The package Microbiome R was used to estimate alpha‐diversity measures and microbial composition (Lahti et al., 2017), beta‐diversity was inferred through Bray–Curtis distances (McMurdie & Holmes, 2013), and statistical differences in microbial taxa at different fermentation times (0, 8 and 24 h) were calculated using ANCOM (Lin & Peddada, 2020). Only taxa showing *p*adj values lower than 0.05 were considered.

### Short‐chain fatty acids analysis

Acetate, propionate, isobutyrate, butyrate, isovalerate and valerate were quantified by gas chromatography (GC) system coupled to a flame injection detector (FID) and a mass spectrometry (MS) 5973 N detector (Agilent), as previously described (Calvete‐Torre et al., 2022; Salazar et al., 2008). Briefly, samples from faecal fermentations were centrifuged (10 min at 10,000 *g*) and 250 μL of supernatants were mixed with 300 μL methanol, 50 μL internal standard solution (2‐ethylbutyric 1.05 mg/mL) and 50 μL 20% v/v formic acid. Following centrifugation of the mixtures (10 min at 10,000 *g*) supernatants were used for injection into the above described GC system.

## RESULTS

### Ubiquity of *bpeM*

**FIGURE 1:**
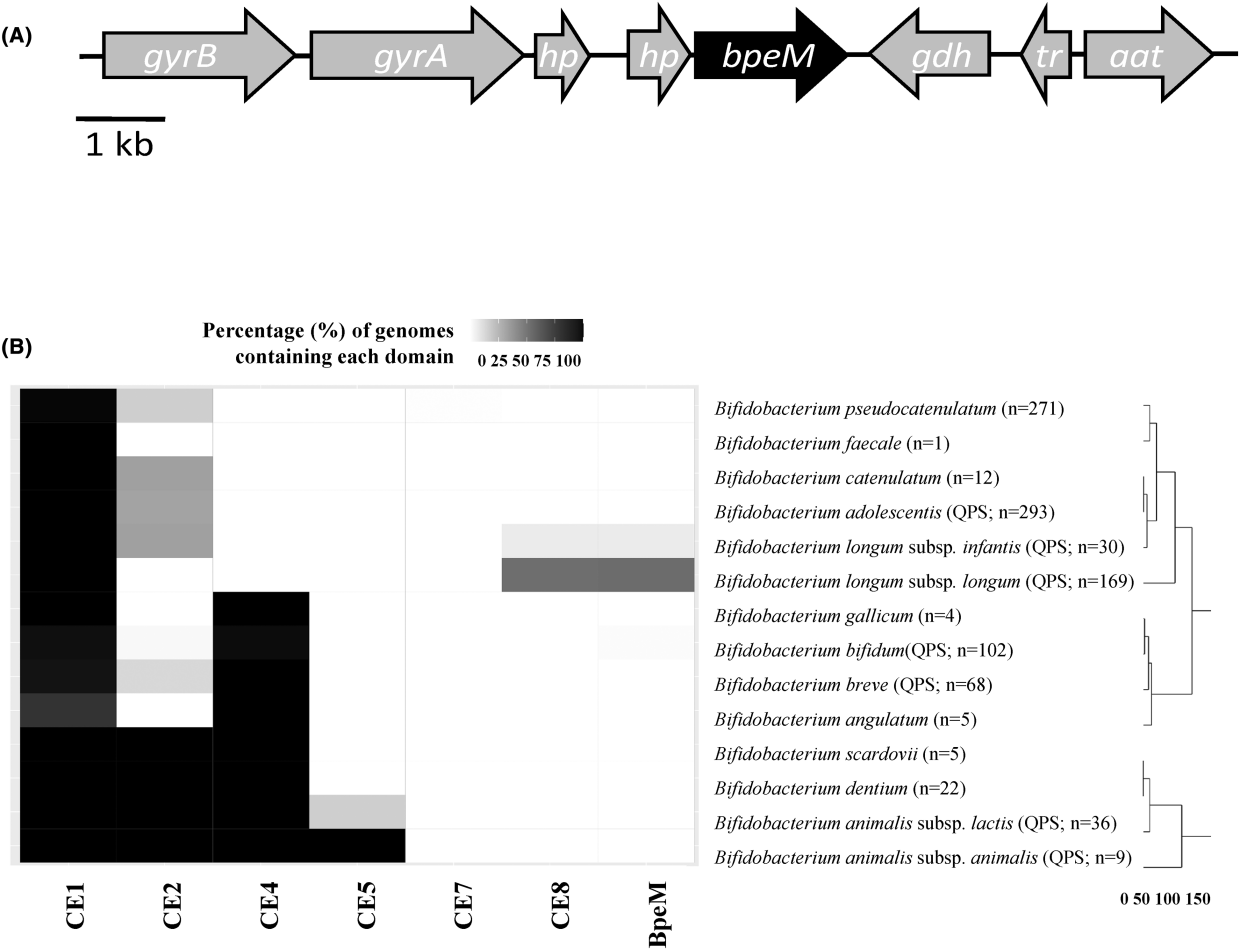
(A) Genetic context of *bpeM* in the genome of *Bifidobacterium longum* LMG13197. *gyrB*, DNA gyrase subunit B; *gyrA*, DNA gyrase subunit A; *hp*, hypothetical protein; *gdh*, glutamate dehydrogenese; *tr*, transcriptional regulator; *aat*, aspartate aminotransferase. (B) Ubiquity of *bpeM* homologues in different bifidobacteria species. Heatmap showing the presence of carbohydrate esterase (CE) domains (indicated as grey and black cells) in the reference genomes and genome assemblies (*n* = 1027) of 14 *Bifidobacterium* species that may be present in human gut microbiota. Functional domains considered comprise xylan and acetyl xylan esterase domains CE1, CE2, CE4, CE5 and CE7, pectin methylesterase domain CE8 and BpeM domain. The percentage (%) of reference genomes and genome assemblies from each species containing each functional domain is shown. Enzyme functional domains showing coverage values higher than 0.95 were annotated. Codes corresponding to the CAZy family of each enzyme have been assigned. More information about these families can be found in the Carbohydrate‐Active enZYmes Database (CAZy). Similarities between *Bifidobacterium* species are also illustrated in a dendrogram and expressed as distances between their characteristic enzyme profiles calculated by the complete linkage method (vertical axis). Some of these species have the Qualified Presumption of Safety (QPS) status.

In a previous study, the genome of the strain *B. longum* LMG13197 was found to harbour a gene, *bpeM*, homologue to pectin esterases described in intestinal bacteria (Gene ID: 69577261, NCBI accession code: BLLJ_RS00045) (Blanco et al., 2020). The fact that *B. longum* has an enzyme with pectinolytic potential caught our attention, since this bacterium does not have an enzymatic arsenal that could indicate that it can degrade pectin by itself. Besides, pectin esterases are frequently located in PULs, described in microorganisms of different origins, including several members of the gut microbiota, but *B. longum* lacks any PUL or any additional genes that may be involved in pectinolytic activities (Figure 1A,B). In fact, to date no bifidobacterial species have been deemed capable of metabolizing pectin (Kelly et al., 2021), although some pectic fractions were reported to promote bifidobacterial groups in faecal fermentations (Gullón et al., 2011; Mao et al., 2019). Bacterial pectin esterases are known to initiate pectin metabolization. Hence, the presence of an isolated pectin esterase in the genome of non‐pectinolytic bifidobacteria likely suggests that the de‐esterification activity could prepare the substrate to be metabolized by other bacteria that, indirectly, can provide a benefit to the enzyme‐producing strain. On the other hand, the ubiquity of the *bpeM* structural gene and other CE domains across bifidobacteria were investigated through a hierarchical clustering modelling (Figure 1B). Bioinformatics analysis showed that BpeM belongs to the carbohydrate esterase family 8 (CE8), composed of pectin methylestarases (EC 3.1.1.11). Domain analysis in a selection of the most common bifidobacterial species in the human gut (*B. longum*, *B. breve*, *B. bifidum*, *B. animalis, B. adolescentis*, *B. angulatum*, *B. catenulatum*, *B. dentium*, *B. faecale*, *B. gallicum*, *B. pseudocatenulatum* and *B. scardovii*) revealed the presence of the xylan and the acetyl xylan esterase domains CE1, CE2, CE4, CE5 and CE7 in the majority of *Bifidobacterium* species under study. The unspecific CE1 domain was present in the genome sequences retrieved from public repositories while the CE5 was characteristic of *B. animalis*. Interestingly, the pectinmethylesterase domain CE8 and the *bpeM* structural gene were characteristic of *B. longum*. In this regard, *bpeM* homologues were annotated in approximately 50% genome sequences of *B. longum* subsp. *longum* (*n* = 169) and approximately 5% genome sequences of *B. longum* subsp. *infantis* (*n* = 30).

To further investigate the distribution of *bpeM* homologues in *B. longum*, a phylogenetic tree of *B. longum* subsp. *longum* genomes (*n* = 169) was generated using PhyloPhlAn v.3.0.67 software (Figure S2). No major differences were observed between *B. longum* subsp. *longum* genomes showing *bpeM* homologues (*n* = 91) and those lacking this pectin esterase domain (*n* = 78), *bpeM*‐genomes being present across all branches of the phylogenetic tree. It should be noted that no transposase domains were identified in the same genomic region so no sign of horizontal gene transfer could be determined. In addition, the closest homologues to this pectin esterase domain in non‐bifidobacterial taxa determined by protein–protein BLAST correspond to a pectinesterase family protein (GenBank MDU5925455.1) from *Finegoldia magna* (99.69% identity percentage) and a hypothetical protein KHY68_06605 (GenBank MBS5233824.1) from *Collinsella* sp. (99.62% identity percentage).

### Purification, factorial modelling and experimental demonstration of the enzymatic activity of BpeM

Expression of the recombinant BpeM protein was achieved by nisin induction in actively growing cells of *L. lactis* NZ9000 harbouring the plasmid pNGBpeM. Control cells, harbouring the empty vector, or cells before induction did not show any apparent presence of the protein in the *L. lactis* extracts. BpeM purification from cell extracts by a single affinity chromatography step with a nickel chelate affinity resulted in a protein more than 95% pure as determined by densitometric and Western blot analysis (Figure S3A,B).

**FIGURE 2:**
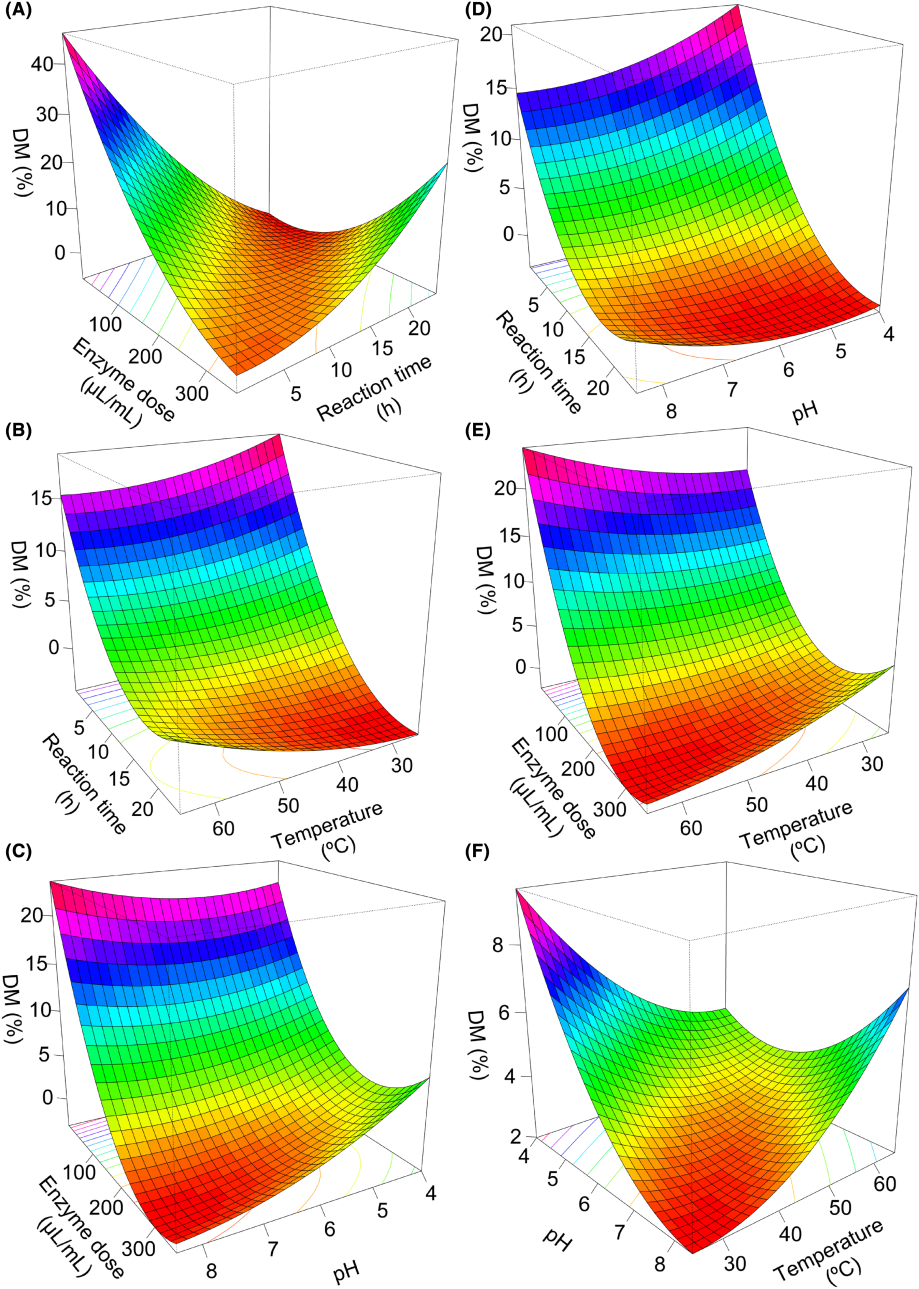
Surface plot illustrating the influence of reaction conditions on the degree of methyl‐esterification (DM) of pectin: (A) reaction time (h) and enzyme dose (μL/mL pectin), (B) reaction time (h) and temperature (°C), (C) enzyme dose (μL/mL pectin) and pH, (D) reaction time (h) and pH, (E) enzyme dose (μL/mL pectin) and temperature (°C), (F) pH and temperature (°C).

Mathematical modelling of the activity of the purified enzyme was performed to gain a better understanding of the pectin de‐esterification process it catalyses. To this end, a factorial experimental design consisting of a set of 43 activity assays was used (Table S2). In this regard, the effect of four independent variables (reaction time, h; pH; temperature, °C; and enzyme dose, μL/mL pectin) on pectin DM was investigated. The experimental design was analysed by RSM method (i.e. subjected to multiple nonlinear regressions to achieve the coefficients of a second order polynomial model). As a result, RSM equations describe the mathematical associations between reaction conditions and pectin DM after the enzymatic reaction. The quality of the fit of the polynomial model equation (i.e. RSM optimization) was expressed by the coefficient of determination R‐squared (*R* ^2^) and the adjusted *R*‐squared (*R* ^2^adj). High *R* ^2^ and *R* ^2^adj were achieved, 0.926 and 0.889 respectively. In addition, error rates of the model were below 5%, highlighting the quality of the model. High enzyme dose values (Figure 2A,C,E) and prolonged reaction times (Figure 2B,D,E) contributed to low DM. Complex interactions between enzyme dose and reaction time were also determined, so reaction conditions must be carefully selected to achieve specific DM values (Figure 2A). In general, reaction temperature and pH values did not exert a great influence on the model (Figure 2F). Then, this mathematical model was used to generate combinations of independent variables that allow obtaining pectin with specific DM values: 10% DM (reaction conditions: time 4.0 h, pH 6.2, temperature 45°C, enzyme dose 150 μL/mL pectin), 20% DM (reaction conditions: time 4.0 h, pH 6.2, temperature 45°C, enzyme dose 80 μL/mL pectin) and 40% DM (reaction conditions: time 1.0 h, pH 6.2, temperature 45°C and enzyme dose 17 μL/mL pectin). These predictions were experimentally validated as, by using the reactions conditions specified above (predicted by the model), we were able to efficiently demethylate native apple high‐methoxyl pectin (DM 69%) in a well‐controlled manner, yielding pectin with DM of 12%, 22% and 42% respectively. Besides, the purified enzyme demonstrated stability upon 1–10 weeks of enzyme storage, irrespectively of the storage temperature (4°C or −20°C) (Figure S4).

### Modification of the prebiotic activity of pectin

**FIGURE 3:**
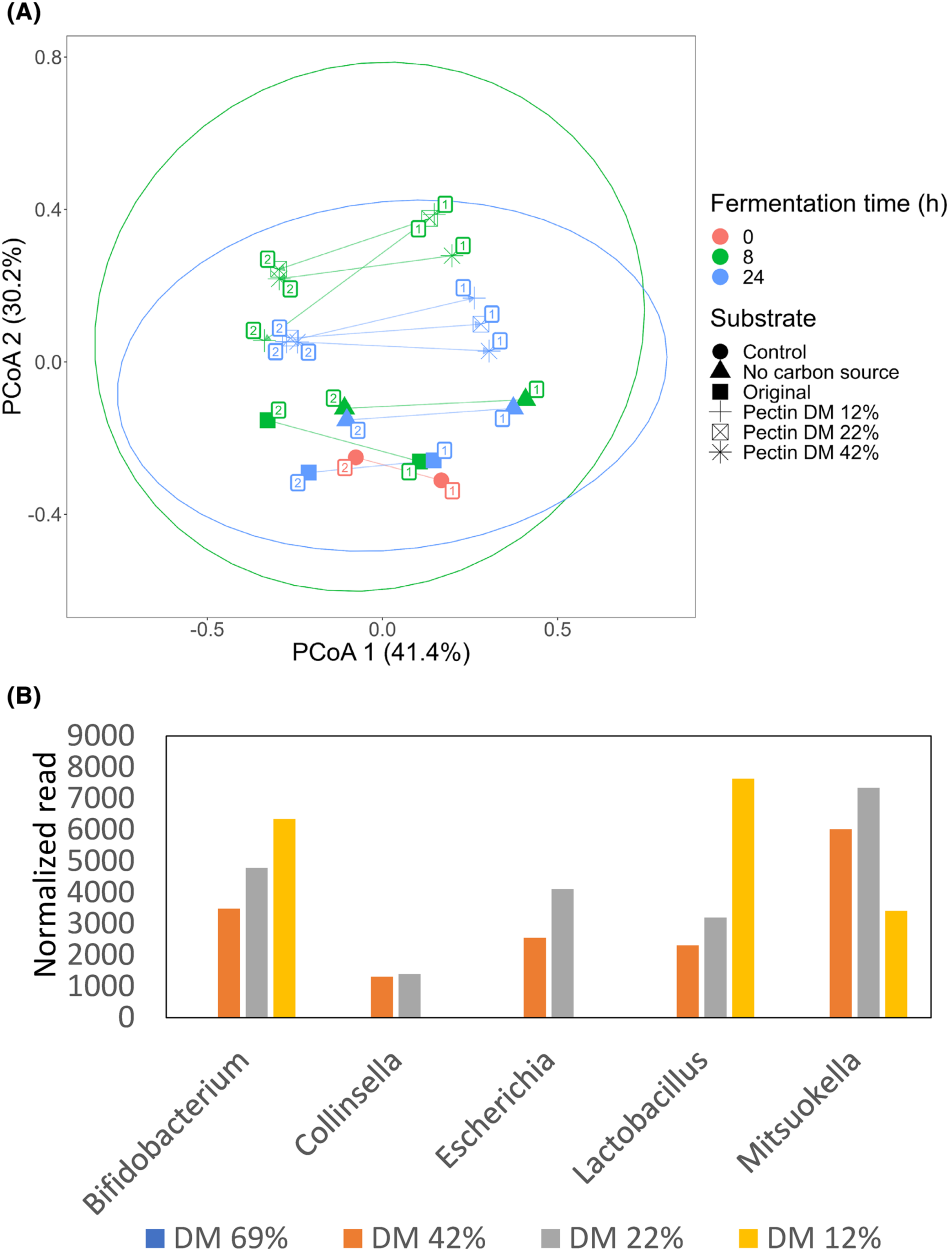
(A) Principal coordinates analysis (PCoA) of faecal fermentations of original pectin (Original) and partially de‐esterified pectin showing degrees of methyl‐esterification (DM) of 12%, 22% and 42% using samples collected from healthy donors. Pectin fractions were compared to controls (where no additional carbon source was added). Samples are labelled according to the substrate type and fermentation time (0, 8 and 24 h). PCoA, principal coordinate. The percentage of variance explained by each PCoA is indicated in the axis. Donor numbers corresponding to each faecal fermentation experiment are indicated in squares. (B) Fold increase (in number of normalized 16S rRNA gene reads) of five specific bacterial genera after 24 h of faecal fermentation with the different pectin variants. Read counts at t 0 (before the cultivation) were normalized to 1. Blue, native pectin (69% DM); orange, pectin with 42% DM; grey, pectin with 22% DM; yellow, pectin with 12% DM.

The capability to modulate gut microbial species of native apple pectin (DM = 69%) and pectin partially de‐esterified by BpeM action, showing DM of 12%, 22% and 42%, was investigated through mixed faecal fermentation cultures, by using samples from healthy donors. Alpha diversity index Chao1 of faecal homogenates (corresponding to the initial fermentation time, 0 h) was 82.5 ± 2.1, showing a small amount of variability between faecal pools. Beta‐diversity coefficients based on Bray–Curtis dissimilarity metric of samples fermented at 8 h were significantly higher (*p* > 0.05) than those of samples fermented at 24 h, suggesting more divergent microbial communities at 8 h of fermentation. No statistically significant differences in beta‐diversity coefficients were detected when comparing fermentations with different substrates (data not shown). Principal coordinates analysis (PCoA) based on Bray–Curtis distances discriminated fermentations with partially de‐esterified pectin fractions from those with original non‐modified pectin and from those carried out without additional carbon source (Figure 3A,B). These results highlight differences in the fermentability of substrates that could be attributed to the lower DM of pectin fractions generated by treatment with the BpeM enzyme.

**FIGURE 4:**
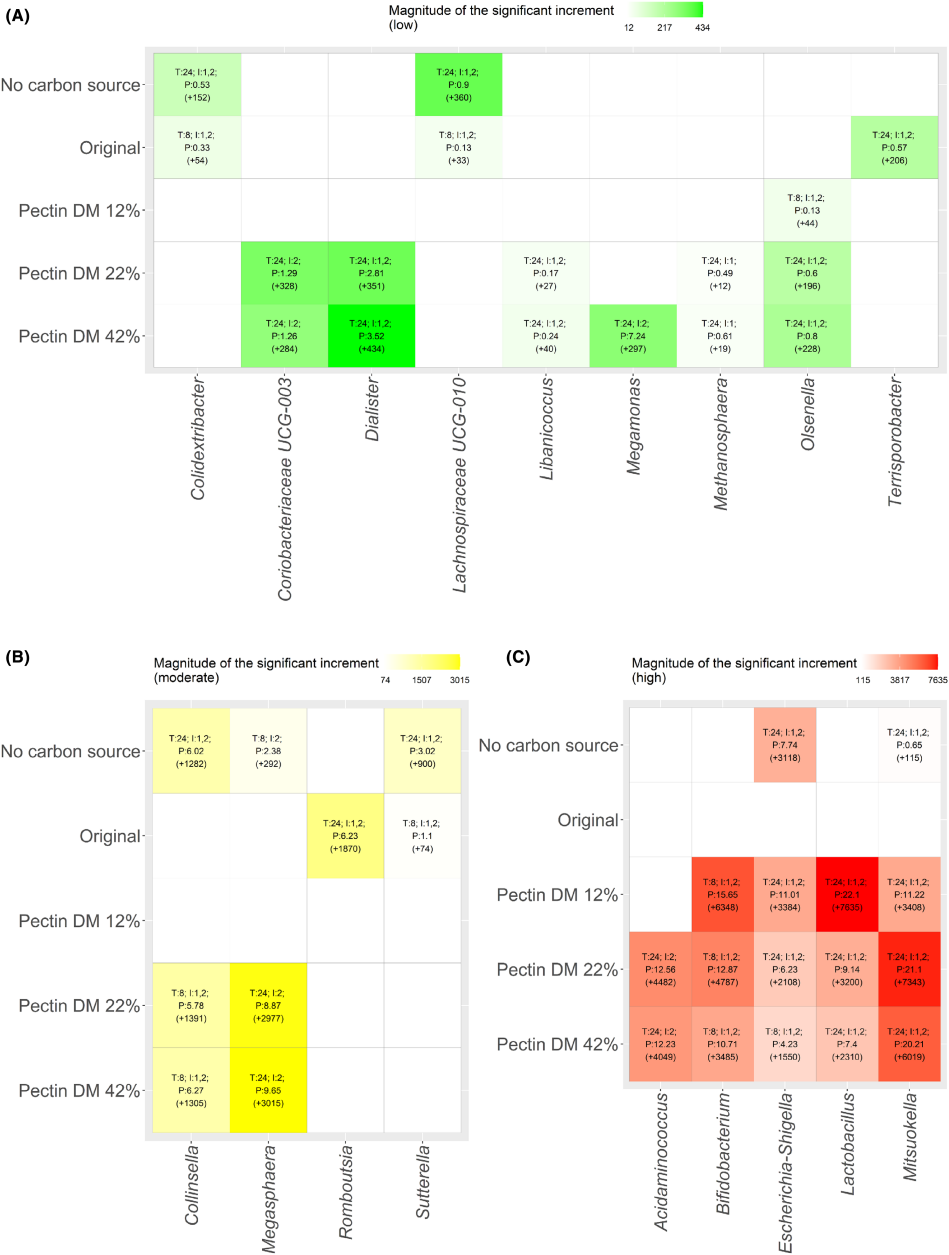
Statistically significant (*p*_adj_ < 0.05) increments in bacterial genera after faecal fermentations of original pectin (Original) and partially de‐esterified pectin showing degrees of methyl‐esterification (DM) of 12%, 22% and 42% using samples collected from healthy donors. These increments were classified into low (A), moderate (B) and high (C) increments compared to the initial fermentation time. Low increment values were comprised between zero and the second quartile. Similarly, moderate increment values were comprised between the second and the third quartiles. Finally, high increment values were comprised between the third and the fourth quartiles. Pectin fractions were compared to controls (where no additional carbon source was added). T: fermentation time at which maximum increment of a specific genus was observed. I: individuals (faecal inoculums) showing the maximum increment of a genus (it should be noted that most significant differences were observed in both faecal inocuolums). P: abundance percentage of a specific genus showing a maximum increment at a given time. Bacterial counts increments are shown in parentheses.

Determination of statistical differences in specific microbial genera among fermentations revealed a total of 29 genera that showed statistically (*p*_adj_ < 0.05) significant increments in their abundances after mixed faecal cultures. Significant increments in 18 of these taxa occurred in fermentations incorporating pectin samples. Microbial increments were classified into low (values between zero and the second quartile), moderate (values between the second and the third quartiles) and high (values between the third and the fourth quartiles) (Figure 4). Genera showing low increments included *Coriobacteriaceae* UCG‐003, *Libanicoccus*, *Methanosphaera* and *Olsenella* that were promoted by pectin with medium DM values (22%–42%). *Olsenella* was also stimulated by low‐DM pectin (DM 12%) while *Megamonas* was only promoted by pectin with DM 42% (Figure 4A). With regard to those genera showing moderate increments, *Dialister* was stimulated only by pectin showing DM of 22% and 42% (Figure 4B). Among those taxa showing the highest increments, *Bifidobacterium* and *Lactobacillus* were selectively stimulated by de‐esterified pectin, the highest increments being detected with the pectin with the lowest DM values (12%) (Figure 4C). Worthy of note, demethylated pectin, as compared to native pectin, promoted a stronger increase of genera associated with beneficial effects. Indeed, some bacterial groups, including *Lactobacillus*, *Bifidobacterium* and *Collinsella*, increased their populations more than three orders of magnitude after incubation with demethylated pectin (Figures 3B and 4A–C).

**FIGURE 5:**
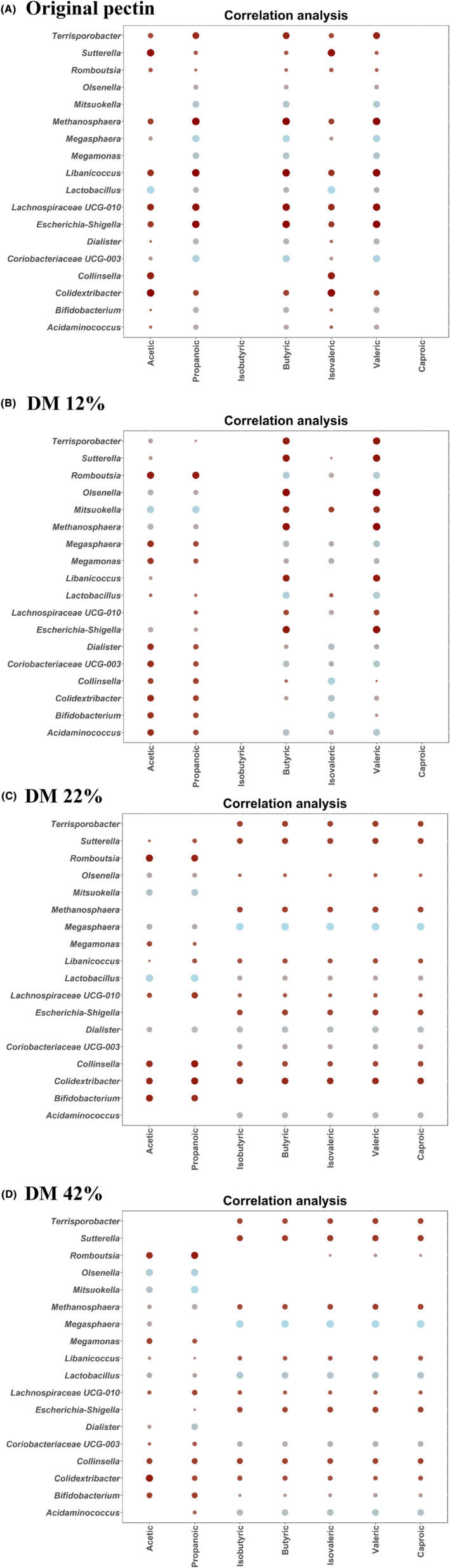
Correlogram showing the associations between short‐chain fatty acids (SCFAs) production and microbial genera showing statistically significant (*p*_adj_ < 0.05) increments after faecal fermentations of original pectin (A) or partially de‐esterified pectin showing degrees of methyl‐esterification (DM) of 12% (B), 22% (C) and 42% (D) using samples collected from healthy donors. Blue and red dots indicate positive and negative correlations expressed as Pearson correlation coefficients. Colour intensity is in proportion to magnitude.

Correlation analysis between SCFAs production and the microbial genera promoted by faecal fermentation of original and partially de‐esterified pectin (Figure 5A–D) revealed positive correlations between a wide range of genera and SCFA levels. Remarkably, positive associations between specific SCFAs and producer taxa generally presented a higher correlation coefficient in the fermentations exhibiting higher promotion of the corresponding taxa. For instance, *Mitsuokella*, a propionate producer, correlated positively with acetate and propionate, the correlation being stronger in demethylated pectin, in which a stronger promotion of this genera was observed. Other positive correlations with SCFAs include *Acidaminococcus* and *Megasphaera* (specially in fractions showing 22–42% DM corresponding to the fermentations with stronger promotion of these taxa).

## DISCUSSION

This study aimed at evaluating whether a predicted pectin methylesterase from a non‐pectinolytic bifidobacterial strain may efficiently demethylate pectin, and at exploring whether this may affect how the modified pectin was fermented by faecal cultures. One of the most striking findings of our work is that apple pectin demethylation has a profound impact on its ability to modulate bacterial taxa in faecal cultures. Reduced fermentation efficiency of highly DM pectin has been reported (Tian et al., 2016, 2017), which is consistent with the microbial modulation detected in our study, and with the lowest production of SCFAs observed in fermentations with highly methoxylated pectin. In relation to SCFAs production, our results demonstrated lower concentrations of SCFAs produced with pectin with the lower DM (12%), as compared with pectin with 22% and 42% DM. Native pectin (with 69% DM) yielded the lowest amounts of SCFAs after the fermentation period, indicating that high‐methoxyl esterification protects the native pectin against microbial degradation processes in which SCFA producers of the microbiota could be involved. However, these results contrast with prior works that have reported that in vitro fermentation of highly‐methoxyl (HM) pectin tend to result in a higher production of propionate in detriment of acetate and butyrate production, likely as a result of the microbial populations modulated (Larsen et al., 2019). A rationale for this difference in relation to the production of SCFAs in HMP may be related to differences in the faecal microbiota samples used as inoculum, as well as to differences in other chemical and structural characteristics of the pectin samples under investigation (Larsen et al., 2019). Notwithstanding, prior works comparing HMP vs. low‐methoxylated (LM) pectin, have generally used pectin from different origins that differ in various attributes apart from their DM, and hence their results may also be influenced by differences in other pectin attributes different from DM.

To the best of our knowledge the work herein presented is the first report showing that controlled demethylation of a HM pectin, caused by a pectin methylesterase of a gut bacterium, severely impacts its fermentability by gut microbial species, resulting in selective enrichment of commensal species belonging mainly to lactobacilli and bifidobacterial groups. In this sense, there are very few publications on pectin methyl‐esterases. A genomic and proteomic analysis of *Monoglobus pectinilyticus*, a pectin‐degrading specialist bacterium from the human colon, showed that it possesses disproportionally large numbers of genes encoding CEs (eight putative methyl‐pectin esterases and four pectin acetyl esterases). These enzymes presumably provide a competitive advantage by removing methyl‐ and acetyl groups to facilitate rapid access by pectin lyases (Kim et al., 2019). Recently, a new family of pectin methylesterases essential for the metabolism of the complex pectin rhamnogalacturonan‐II was described in *Bacteroides thetaiotaomicron* (Duan et al., 2020). Apart from the information mentioned above, the role played by these types of enzymes in shaping the fermentability (prebiotic capacity) of pectin and hence, determining its gut microbial ecosystem modulation capacity, is unknown.

Chemical and structural features of fibre are key determinants of their biological properties and explain the variation observed in the prebiotic properties of related, yet structurally different fibre. Metabolism of vegetable polysaccharides in the gut is initiated by some bacteria producing endo and exo‐acting GHs that break glycosidic linkages to release carbohydrates with lower molecular weight. For example, in the arabinoxylan metabolism, many of the products resulting from this activity, including oligomers derived thereof, arabinose and xylose can be further metabolized by accompanying members of the microbiota that take advantage of the released oligosaccharides to complete the degradation of the substrates (Feng et al., 2018; Pereira et al., 2021). Evidence suggests that the DM of complex fibre is one of the characteristics that impact their bioactivity and prebiotic properties, affecting their accessibility by some bacteria. Fibre esterification can hinder GH accessibility and the esterification degree of fibre can decisively determine the specific taxa that can initiate their fermentation within the gut ecosystem, and the metabolic cooperative networks established within the microbiome. This ultimately conditions the metabolic end products of the fermentation and health‐promoting effects of different fibre structures (Elshahed et al., 2021). In this regard, it was shown that pectin from different sources modulate the faecal microbial population depending on the DM; LMP pectin being generally fermented faster than HM pectin in vitro (Cui et al., 2020; Larsen et al., 2019) and in vivo (Dongowski et al., 2002; Tian et al., 2016, 2017; Wu et al., 2019). Remarkably, among the bacteria whose populations increase on fermentations with low DM pectin, we found lactobacilli and bifidobacteria, whose administration in humans has been associated with a wide range of beneficial effects (Hidalgo‐Cantabrana et al., 2017; Salvetti & O'Toole, 2017). Finally, some studies have evidenced that in vitro synthesis of prebiotics with enzymes from gut bacteria, can result in the generation of substrates that are fermented preferentially by the corresponding enzyme‐producing strain or closely related species. This situation has been demonstrated for GOS produced with beta‐galactosidases derived from bifidobacteria (Tzortzis et al., 2005). Further research is necessary to evaluate the possibility of exploiting CEs from gut microbiota members to re‐programme the pattern of fermentation of pectic prebiotics in the gut ecosystem, and to decipher the metabolic networks established in the presence of the demethylated pectin, that lead to promoting non‐pectinolytic bacterial groups. This would pave the way to the synthesis of smart prebiotics, capable to efficiently promote target species in the gut microbiota upon consumption.

Some limitations of the present investigation include the reduced number of cultures performed, and the fact that pooled faecal inocula were used in the fermentations. The later fact may have introduced certain biases in the fermentation patterns described in this investigation. These facts limit our capability to discern the metabolic cooperation networks involved in fermenting pectin samples with different DM in the gut ecosystem, as well as to detect interpersonal variation in the fermentation of the different pectin variants tested. Besides, batch fermentations with no control of pH or nutrients addition during the fermentation, could have selectively favoured some microbial groups, masking slow fermenters. Another point of consideration may be related to possible secondary activities of the BpeM. While methyl pectinesterases have not been reported to affect other pectin characteristics, such as molecular weight or pectin branching, structural confirmation of all relevant characteristics that can affect pectin fermentability would help dilucidated the molecular mechanisms favouring the fermentability of low DM pectin by specific gut commensal groups, and confirm the role of DM modification in affecting pectin fermentability. Regardless of these limitations, the present study provides consistent and reproducible results pointing to a relevant role of BpeM to facilitate fermentability of pectin by bifidobacteria and lactobacilli when present in mixed faecal cultures. Further investigation should deep into the possibility of exploiting this activity to programme the prebiotic properties of pectin, and to decipher the metabolic networks established in the gut ecosystem in the presence of non‐pectinolytic bacteria encoding *bpeM*.

In summary, as research in the field evolves, the prebiotic concept is expanding to encompass chemical structures different from the traditional prebiotic oligosaccharide structures. Besides, emerging research is focusing on the personalization of dietary interventions based on individual differences in microbiota and the predicted responses to given food ingredients and dietary patterns. For this reason, design of foods and ingredients specifically tailored to particular microbiota components is attracting increasing scientific attention as a mean to ameliorate health conditions associated to microbiome imbalances. Rationale design of such ingredients requires deep knowledge on microbiome structure–function relationships, so as to provide through diet ingredients capable to be transformed into bioactives, and/or to reconfigure the microbiota conformation in a beneficial way. Modifying the DM of pectin in a well‐controlled, affordable and predictable manner is an appealing alternative that can contribute to enhance the prebiotic properties of pectin from different sources. This may aid to diversify the range of microbiome‐acting ingredients.

## AUTHOR CONTRIBUTIONS

**Inés Calvete‐Torre:** Formal analysis; investigation; writing – original draft. **Carlos Sabater:** Formal analysis; software; visualization; writing – original draft. **Nerea Muñoz‐Almagro:** Formal analysis; methodology. **Ana Belén Campelo:** Investigation. **F. Javier Moreno:** Conceptualization; methodology; writing – review and editing. **Abelardo Margolles:** Conceptualization; project administration; resources; supervision; writing – original draft; writing – review and editing. **Lorena Ruiz:** Conceptualization; project administration; supervision; writing – original draft; writing – review and editing.

## FUNDING INFORMATION

We thank the projects MICINNAGL2016‐78311‐R, RTI2018‐095021‐J‐I00, PID2021‐123862OB‐100 and PID2022‐141737OB‐I00, funded by MCIN/AEI/ 10.13039/501100011033/ and by ERDF A way of making Europe; and AYUD‐2021‐50910 from the autonomic Government of Principado de Asturias (FICYT, supported by FEDER). Carlos Sabater was the recipient of a Juan de la Cierva‐Formación postdoctoral contract from MICINN, the Spanish Ministry of Science and Innovation (FJC2019‐042125‐I).

## CONFLICT OF INTEREST STATEMENT

There are no conflicts of interest to declare.

## Supporting information


Figure S1:

## Data Availability

Sequences generated in this work are publicly available in the SRA archive under accession number PRJNA1030193.
