# Supplementary material for: A methyl esterase from Bifidobacterium longum subsp. longum reshapes the prebiotic properties of apple pectin by triggering differential modulatory capacity in faecal cultures
Source: Microb Biotechnol. 2024 May 9;17(5):e14443. doi: 10.1111/1751-7915.14443 (PMC11081426; doi:10.1111/1751-7915.14443)

**Supplementary Table 1.** Primers used for amplification of the *bpeM* encoding gene for subsequent cloning into pNZE8048.

| **Primer name** | **Sequence** |
| --- | --- |
| **bpeM-fw** | 5´-CGCTGC*TC****ATG****A*ACGCTGGAACCATC-3´ |
| **bpeM-rev** | 5´-CTCATG*TCTAGA***TCA**ATGGTGATGGTGATGGTGATGGTGATGGTGCTGCTGGCGATTGCGG-3 |

BspHI and XhoI sites appear ictalized in bpeM-fw and bpeM-rev, respectively

Translational start codon and stop codon appear in bold letters

His-tag encoding sequence is underlined

**Supplementary Table S2.** Factorial experimental design consisting of set of 43 activity assays under a set of conditions. These activity assays were selected to investigate the effect of four independent variables (reaction time, h; pH; temperature, ⁰C; and enzyme dose, µL/mL pectin) on pectin degree of methylesterification (DM) (%) via mathematical modelling (See **Fig. 2**).

| **Test** | **Time (h)** | **pH** | **T (⁰C)** | **Enzyme (µL/mL pectin)** |
| --- | --- | --- | --- | --- |
| **1** | 17.1 | 7.1 | 53.3 | 265.0 |
| **2** | 7.3 | 7.1 | 53.3 | 265.0 |
| **3** | 17.1 | 5.3 | 53.3 | 135.0 |
| **4** | 12.2 | 6.2 | 45.0 | 200.0 |
| **5** | 7.3 | 5.3 | 53.3 | 135.0 |
| **6** | 7.3 | 5.3 | 36.7 | 135.0 |
| **7** | 17.1 | 7.1 | 36.7 | 265.0 |
| **8** | 7.3 | 7.1 | 53.3 | 135.0 |
| **9** | 12.2 | 6.2 | 45.0 | 200.0 |
| **10** | 12.2 | 6.2 | 45.0 | 200.0 |
| **11** | 7.3 | 7.1 | 36.7 | 265.0 |
| **12** | 12.2 | 6.2 | 45.0 | 200.0 |
| **13** | 17.1 | 7.1 | 53.3 | 135.0 |
| **14** | 12.2 | 6.2 | 45.0 | 200.0 |
| **15** | 17.1 | 5.3 | 36.7 | 135.0 |
| **16** | 17.1 | 5.3 | 36.7 | 265.0 |
| **17** | 12.2 | 6.2 | 45.0 | 200.0 |
| **18** | 17.1 | 5.3 | 53.3 | 265.0 |
| **19** | 7.3 | 5.3 | 53.3 | 265.0 |
| **20** | 7.3 | 5.3 | 36.7 | 265.0 |
| **21** | 7.3 | 7.1 | 36.7 | 135.0 |
| **22** | 17.1 | 7.1 | 36.7 | 135.0 |
| **23** | 12.2 | 4.0 | 45.0 | 200.0 |
| **24** | 24.0 | 6.2 | 45.0 | 200.0 |
| **25** | 12.2 | 6.2 | 45.0 | 43.0 |
| **26** | 0.4 | 6.2 | 45.0 | 200.0 |
| **27** | 12.2 | 6.2 | 65.0 | 200.0 |
| **28** | 12.2 | 6.2 | 25.0 | 200.0 |
| **29** | 12.2 | 6.2 | 45.0 | 357.0 |
| **30** | 12.2 | 8.4 | 45.0 | 200.0 |
| **31** | 2.1 | 6.2 | 45.0 | 51 |
| **32** | 0.7 | 6.2 | 45.0 | 14.5 |
| **33** | 0.7 | 6.2 | 45.0 | 87.5 |
| **34** | 2.1 | 6.2 | 45.0 | 51 |
| **35** | 2.1 | 6.2 | 45.0 | 51 |
| **36** | 2.1 | 6.2 | 45.0 | 51 |
| **37** | 2.1 | 6.2 | 45.0 | 51 |
| **38** | 3.5 | 6.2 | 45.0 | 87.5 |
| **39** | 3.5 | 6.2 | 45.0 | 14.5 |
| **40** | 2.1 | 6.2 | 45.0 | 99.67 |
| **41** | 0.23 | 6.2 | 45.0 | 51 |
| **42** | 2.1 | 6.2 | 45.0 | 2.33 |
| **43** | 3.97 | 6.2 | 45.0 | 51 |

**Supplementary Figure S1.** Phylogenetic tree of *Bifidobacterium longum* subsp. *longum* genomes (n=169). Genome sequences showing bpeM homologues (n=91) and those lacking this pectin esterase domain (n=78) are marked in green and blue, respectively. Phylogenetic tree was generated using PhyloPhlAn v.3.0.67 software


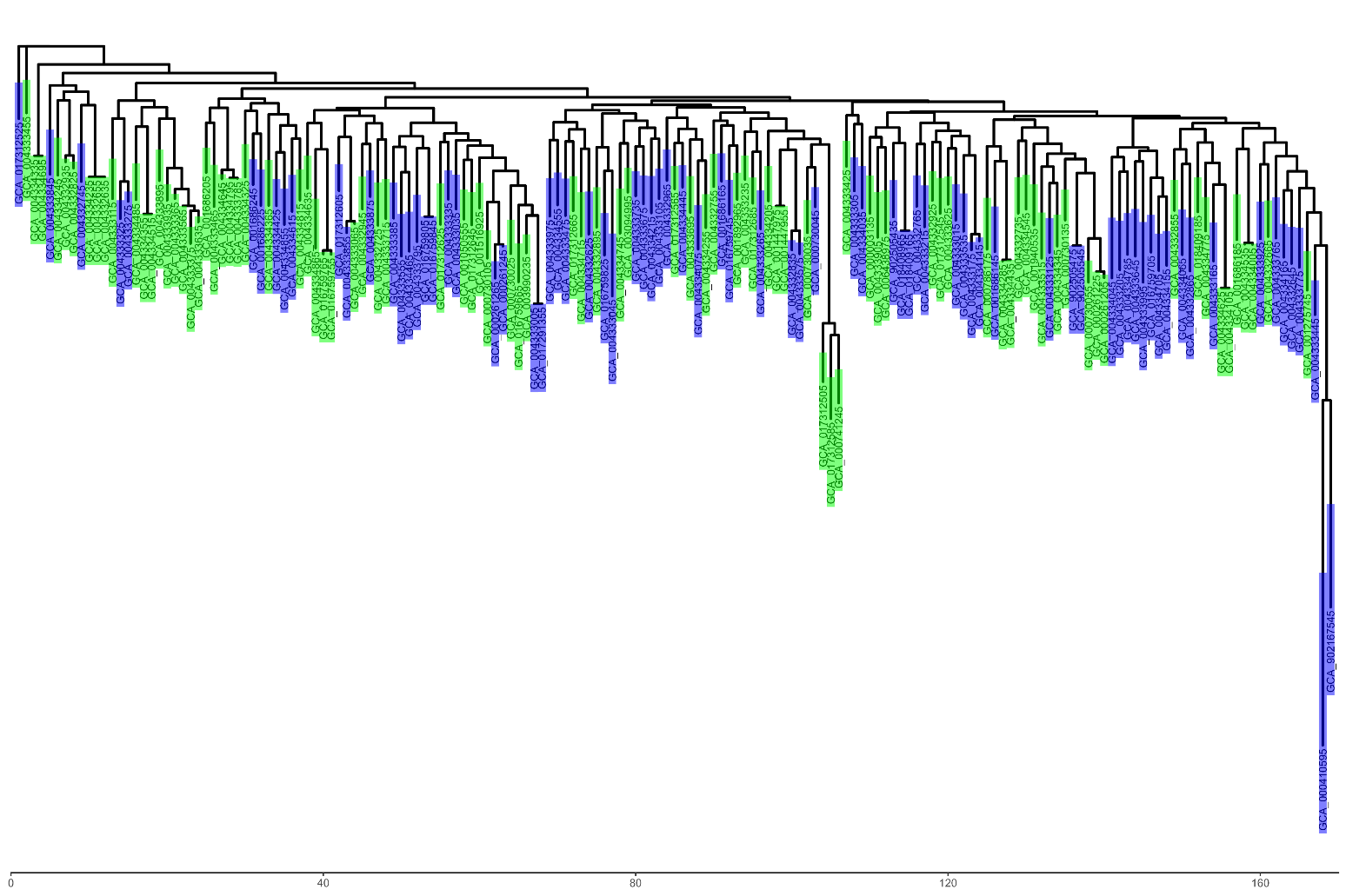


**Supplementary Figure S2.** Representative FTIR spectra of pectin samples with degrees of methyl esterification (DM) of 60.2% (**A**), 44.5% (**B**), 6.9% (**C**) and 0% (**D**) are shown. DM was calculated from the spectra as the peak area at 1747  cm−1 (COO-R) over the sum of the peak areas of 1632  cm−1 (COO−) and 1747  cm−1 (COO-R).

**Supplementary Figure S3.** Purification of the histidine-tagged BpeM. A) Coomassie brilliant blue-stained SDS-PAGE gel showing intermediate steps in the purification procedure. Lane 1, protein marker (molecular masses, in kilodaltons, are indicated); Lane 2, total cell extract of *L. lactis* NZ9000/pNBpeM before nisin induction; Lane 3, total cell extract of *L. lactis* NZ9000/pNBpeM after nisin induction; Lane 4, purified protein eluted from the Ni-NTA column with 250 mM imidazole. B) Western blot analysis of an SDS-PAGE gel containing the same samples as shown in A.


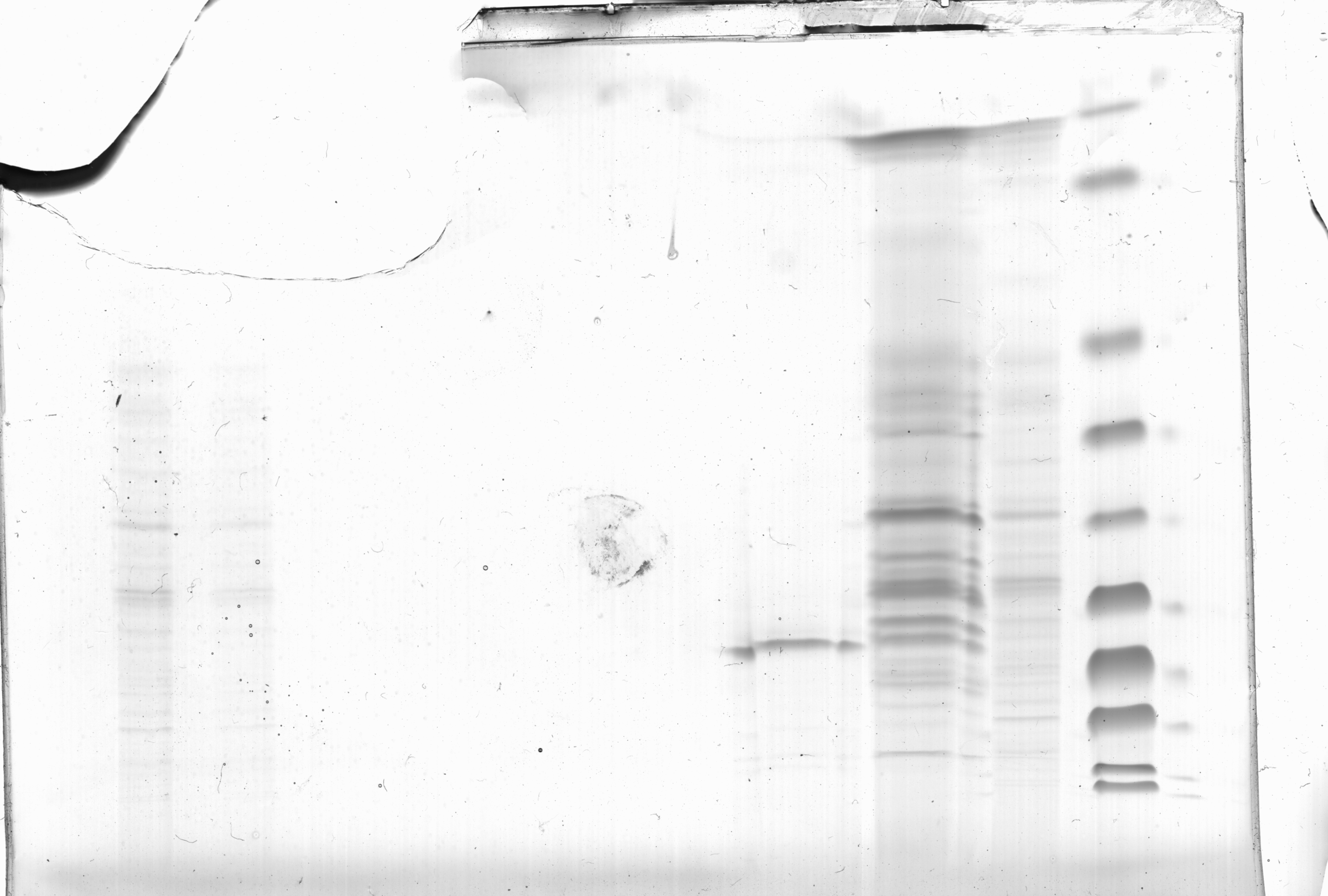


**A**

**B**


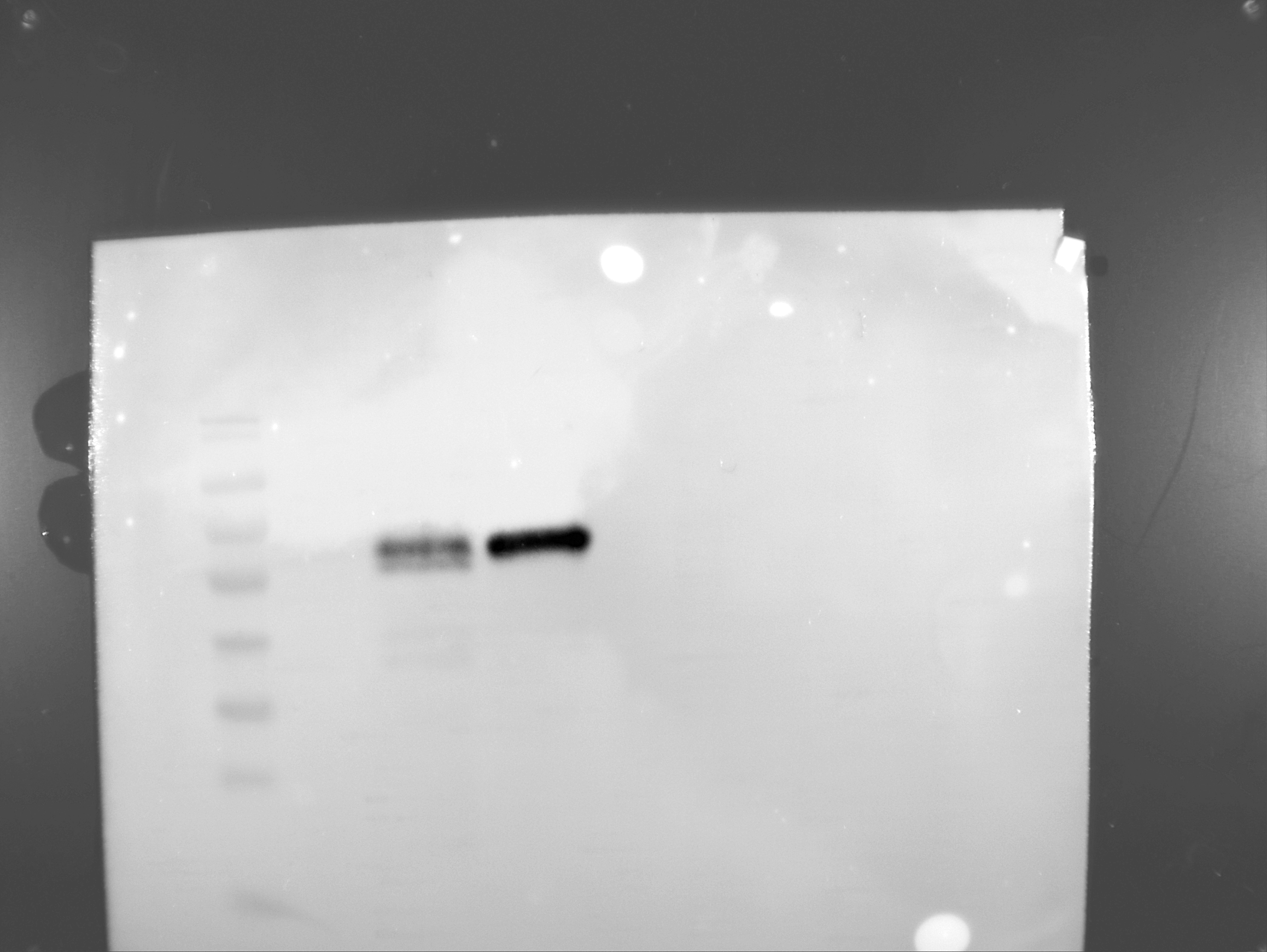


1

1

2

2

3

3

4

4

180

70

15

55

kDa

kDa

130

100

40

25

10

180

70

15

55

130

100

40

25

10

**Supplementary Figure S4.** DM of native apple pectin treated with BpeM after its storage at 4 ºC and -20 ºC. Enzyme activity was tested just after the purification of the enzyme and after 1, 2, 5, 8, 10 and 28 weeks of storage. Mean and standard error of independent biological replicates is represented.


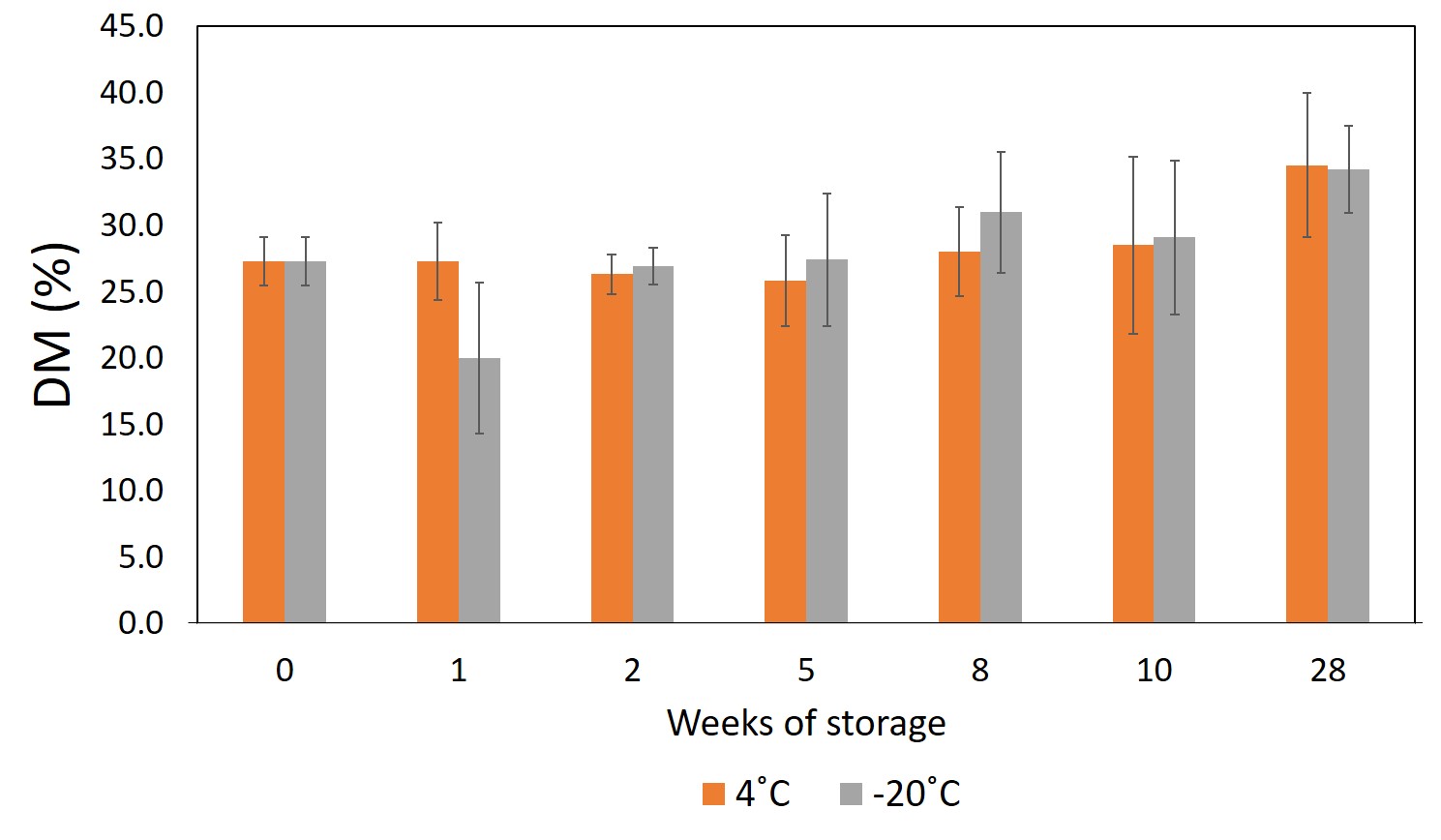


**Supplementary Figure S5.** Beta diversity (A) and cluster (B) analysis of fecal fermentations of fecal fermentations of original pectin (Original) and partially de-esterified pectin showing degrees of methyl-esterification (DM) of 12, 22 and 42% using samples collected from healthy donors. Bray-Curtis dissimilarity method was selected for the calculation. Samples are labelled according to the fermentation time.


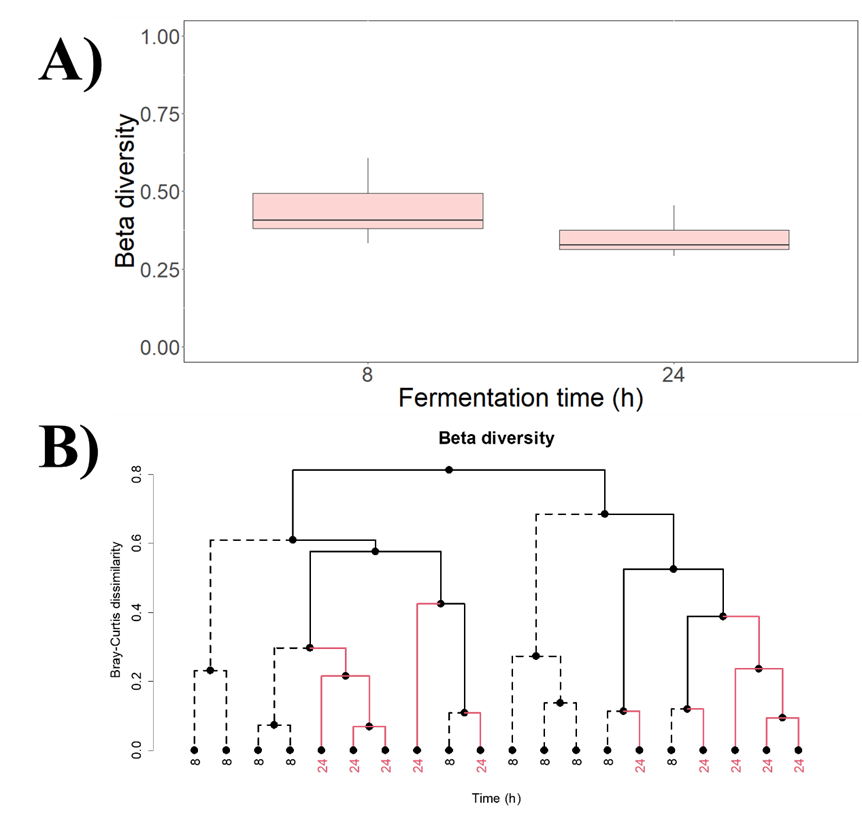

Supplement: Supplementary file 1 — Figure S1: [file MBT2-17-e14443-s001.docx]
